# Genomic characterization and molecular evolution analysis of subtype B and BF recombinant HIV-1 strains among Argentinean men who have sex with men reveal a complex scenario

**DOI:** 10.1371/journal.pone.0189705

**Published:** 2017-12-15

**Authors:** Cintia G. Cevallos, Leandro R. Jones, Maria A. Pando, Jean K. Carr, Maria M. Avila, Jorge Quarleri

**Affiliations:** 1 Instituto de Investigaciones Biomédicas en Retrovirus y Sida (INBIRS), Facultad de Medicina, Universidad de Buenos Aires (UBA), Buenos Aires, Argentina; 2 Consejo Nacional de Investigaciones Científicas y Técnicas (CONICET), Buenos Aires, Argentina; 3 Laboratorio de Virología y Genética Molecular (LVGM), Facultad de Ciencias Naturales y Ciencias de la Salud, sede Trelew, Universidad Nacional de la Patagonia San Juan Bosco, Chubut, Argentina; 4 Lake Erie College of Osteopathic Medicine at Seton Hill, Greensburg, Pennsylvania, United States of America; George Washington University, UNITED STATES

## Abstract

Currently, data on HIV-1 circulating strains among men who have sex with men (MSM) in Argentina is scarce. In South America, the distribution and the prevalence of BF recombinants are dissimilar and exhibit an underappreciated heterogeneity of recombinant structures. Here, we studied for the first time the genetic diversity of HIV-1 BF recombinants and their evolution over time through *in-depth* phylogenetic analysis and multiple recombination detection methods involving 337 HIV-1 nucleotide sequences (25 near full-length (NFL) and 312 partial *pol* gene) obtained from Argentinean MSM. The recombination profiles were studied using multiple *in silico* tools to characterize the genetic mosaicism, and phylogenetic approaches to infer their relationships. The evolutionary history of BF recombinants and subtype B sequences was reconstructed by a Bayesian coalescent-based method. By phylogenetic inference, 81/312 *pol* sequences clustered within BF clade. Of them, 46 sequences showed a genetic mosaic with CRF12_BF-like patterns, including plausible second-generation recombinants. Other CRFs_BF like (CRF17, 28, 29, 39, 42, 44, 47) and probable URFs_BF were less frequently found. Phylogenetic and recombination analyses on NFL sequences allowed a meticulous definition of new BF mosaics of genomic patterns. The Bayesian analyses pointed out quite consistent onset dates for the CRFs_BF clade based on B and F gene datasets (~1986 and ~1991 respectively). These results indicate that the CRFs_BF variants have been circulating among Argentinean MSM for about 30 years. This study reveals, through growing evidence showing the importance of MSM in the dynamics of the HIV-1 epidemic in Argentina, the coexistence of CRF12_BF-like and high diversity of strains exhibiting several BF mosaic patterns, including non-reported URFs that may reflect active clusters as potential intervention targets to hinder HIV-1 transmission.

## Background

The HIV-1 epidemic in Argentina is mostly represented by subtype B, plus a wide variety of recombinant strains between subtypes B and F. Other less frequent HIV-1 subtypes (A, C, F) and recombinant forms (BC, BCF, A2D, and AG) have also been characterized [1–5]. Since the onset of the HIV/AIDS epidemic, men who have sex with men (MSM) have remained a recognized high-risk group for HIV-1 infection. It is well known worldwide that many MSM are at high–risk for infection, hence, HIV-1 prevalence and incidence in MSM are consistently higher than in the general population [6]. According to official data published in 2016 by the Ministry of Health, Argentina, the current prevalence in MSM is between 12 and 15% [7]. The genomic characterization of HIV-1 among Argentinean MSM was performed by phylogenetic inference [3, 8, 9]. Since then, no other evidence has been provided regarding HIV-1 genetic variability associated with BF recombinants among MSM.

In South America, the prevalence rates of BF recombinant sequences, either Circulating Recombinant Forms (CRF_BF) or Unique Recombinant Forms (URF_BF), have been found to be significant. Other CRF_BF have also been described in South America: CRF28_BF and CRF29_BF in Sao Paulo (Brazil) [10]; CRF39_BF and CRF40_BF in Rio de Janeiro (Brazil) [11]; CRF38_BF in Uruguay [12]; and CRF44_BF in Chile [13]. In Argentina after the identification of the CRF12_BF and CRF17_BF in 2001 [14, 15], several studies showed that most BF recombinants circulating locally were closely associated with CRF12_BF or diverse BF recombinants related to it [1, 16–18].

The aim of the present study was to evaluate the heterogeneity of the HIV-1 associated with subtype B and BF recombinants and their evolution over time through *in-depth* phylogenetic and Bayesian coalescent analysis and multiple recombination detection methods among Argentinean MSM. Using near-full length and pol-gene sequences, this study reveals a complex scenario for BF recombinants among MSM where CRF12_BF-like and high diversity of strains exhibiting several BF mosaic patterns -including non-reported URFs- coexist.

## Materials and methods

### HIV-1 nucleotide sequences datasets

Two datasets were constructed involving a total of 337 HIV-1 nucleotide sequences. These include 312 partial *pol* gene sequences (defined from nucleotide 2253 up to 3720 according to HXB-2 numbering) and 25 near full-length (NFL) sequences. These were obtained from 429 HIV-1 seropositive Argentinean men who have sex with men (MSM) recruited in previous studies performed during the period 2000–2009. They are mainly residents of Buenos Aires city and surroundings areas with a median age of 30 years, and most of them were not on antiviral therapy at the time of the studies. As documented previously, the consumption of illicit drugs presented by this population was very infrequent. Blood collection was performed once a written informed consent was obtained as stated in each of the original studies [2, 4, 8, 9, 19]. The methods used for extraction of the DNA and sequencing are described in the respective references. All the sequences used in this work were obtained from projects previously approved by the Ethic Committee from School of Medicine of Buenos Aires University or Ethic Committee of NEXO AC (IRB 00005349). In all cases the consent of each participant, confidentiality of the information as well as laboratory results, were obtained according to international recommendation and current legislation (National AIDS Law No. 23,798 and its regulatory decree) and the recommended international ethical guidelines for epidemiological studies were strictly followed, (CIOMS, Ginebra 2009).

HIV-1 subtype references strains available in Los Alamos Database (http://www.hiv.lanl.gov/content/sequence/HIV/mainpage.html) were used to make alignments with the Argentinean sequences under analysis. The alignments were performed with MAFFT software included in http://www.hiv.lanl.gov/content/sequence/VIRALIGN/viralign.html [20].

### Phylogenetic analysis

Phylogenetic analyses were carried out in order to classify the viruses by HIV-1 subtype or CRFs. Phylogenetic trees were constructed with a GTR nucleotide substitution model among site rate heterogeneity and a proportion of invariable site selected by jModelTest v.2.1.10 [21] using the Maximum Likelihood (ML) method implemented in PhyML v3.0 employing an online web server (http://www.atgc-montpellier.fr/phyml/) [22]. The best SPR and NNI heuristic options were selected. The reliability of tree topologies was assessed by two methods according to dataset, such as bootstrapping using 500 replications (values ≥ 70% were considered significant) for NFL dataset, and approximate likelihood ratios (aLRT) ≥ 0.90 based on Shimodaira-Hasegawa-like procedure were used to assign the subtype/CRF designation of the strains for *pol* dataset [23].

### Detection of recombination events and resistance mutations

The HIV-1 intersubtype recombination patterns were analyzed separately for each sequence by determining the genetic mosaic structure, location of breakpoints, and parental subtypes involved.

For this purpose, several *in silico* methods were selected considering both the type of analysis to be carried out and the nature of the database [24]. Initially, *pol* sequences were analyzed by three different methods or algorithms including: (i) Jumping profile Hidden Markov Model (jpHMM) method. It is based on a pre-calculated multiple alignment of the major HIV-1 subtypes where each one is represented by a profile hidden Markov model further connected by empirical probabilities, allowing the detection of possible recombinants and plausible breakpoints. The HIV-1 subtype B and subtype F have adequate sampling to build well informed profiles offering enough data to construct a good model of sequence variation [25], (ii) Recombination Detection Program (RDP4), a software package for statistical identification and characterization of recombination events in DNA sequences that uses several non-parametric recombination detection methods. Among multiple approaches to identify recombination signals, RDP, BOOTSCAN, GENECONV, MAXCHI, and SISCAN methods were carried out. All methods are described in detail in reference [26]. This tool considers every sequence included in the analysis as a potential recombinant and monitors systematically each sequence triplets or quartets in order to identify those that contain a recombinant as well as those two sequences that could serve as parents while performing a statistical evaluation of recombination signals. The highest acceptable P-value was set to 0.05 and the other parameters were default RDP4 settings. Recombination events detected by at least three different methods (Pvalues of ≤0.05 after Bonferroni correction for multiple comparisons implemented in RDP4) were taken as credible evidence of possible HIV-1 recombination. Lastly, (iii) the recombination analysis using cost optimization (RECCO) was performed. This software computes the number of “savings”, i.e., number of mismatches saved when explaining the read by a recombination event between two viral strains and mutations, rather than by a single strain and mutations only. In this analysis, sequences were defined as recombinant when RECCO assigns a savings value higher than 5 [27]. When conflicting results emerged in recombination events, the criteria were to consider as actual event in which at least two out of the three methods used would match. Only for near full length sequences, bootscanning analysis was also performed separately to detect recombination as implemented in the SimPlot software version 3.5.1 (http://www.med.jhu.edu/deptmed/sray/download/) [28].

The presence of resistance mutations was detected by the Stanford algorithm (https://hivdb.stanford.edu/hivseq/by-mutations/)

### Concordance analysis

This analysis was performed to determine whether the partial *pol* gene sequence contained enough information to define the presence of a BF recombination phenomenon after comparing against the result based on NFL sequences analyses. For this goal, we assessed the degree of agreement by calculating the Kappa of Fleiss index. It was carried out among the 25 near full length sequences and with the *pol* region belonging to them using SPSS version 22. The Kappa degree of agreement was defined according to [29].

### Estimation of the time to the most recent common ancestor (tMRCA) for HIV-1 BF recombinant and subtype B strains

Estimation of tMRCAs and substitution rates were performed by the Bayesian Markov chain Monte Carlo (MCMC) phylogenetic analysis as implemented in the program BEAUTI/BEAST v2 [30] that allows the substitution rate to vary among branches in the tree. The nucleotide substitution models were obtained by the jModelTest program v.2.1.10 [21] and then, they were compared using the Bayes Factor test to find the best fit with Tracer V1.5 [31]. Two clock models were used in this analysis: strict clock model, that assumes a single evolutionary rate for all the lineages, and the uncorrelated lognormal relaxed clock that allows variable evolution rates among lineages within lognormal relaxed. These two clock models were statistically compared using Bayes Factor test to find the best fit. The empirical prior of 2.5 X 10^−3^ substitution per nucleotide site per year was used to analyze the *pol* gene [32]. As recombination could affect molecular clock estimation, we performed an evolutionary reconstruction of subtype B and F fragments from BF recombinant MSM sequences, independently of each other [33]. For subtype B, the region analyzed contained 669 bases in length, located in the partial *pol* gene between positions 3051 and 3719 according to HXB-2 numbering (n = 41). Besides, for subtype F, the region analyzed contained 650 bases in length, located in the partial *pol* gene between position 2253 and 2904 referred to HXB-2 (n = 30). Comparatively, we analyzed 40 partial *pol* B sequences containing 1041 bases in length, located between positions 2253 and 3294 referred to as HXB-2. In order to infer the epidemiological history of our study population, three demographic models were included (constant population size, exponential growth, and Bayesian Skyline) and compared with the Bayes Factor. Finally, three independent Bayesian MCMC runs for 100 million generations were performed. The convergence of parameters was assessed through the effective sampling size, with all parameters for each run with values >100 indicating a sufficient level of sampling. The mean time of the tMRCA and the maximum clade credibility of the phylogenetic trees were then calculated after removing 10% of the samples.

## Results

### Phylogenetic analysis and recombination detection of partial *pol* and near full length sequences

**Fig 1:**
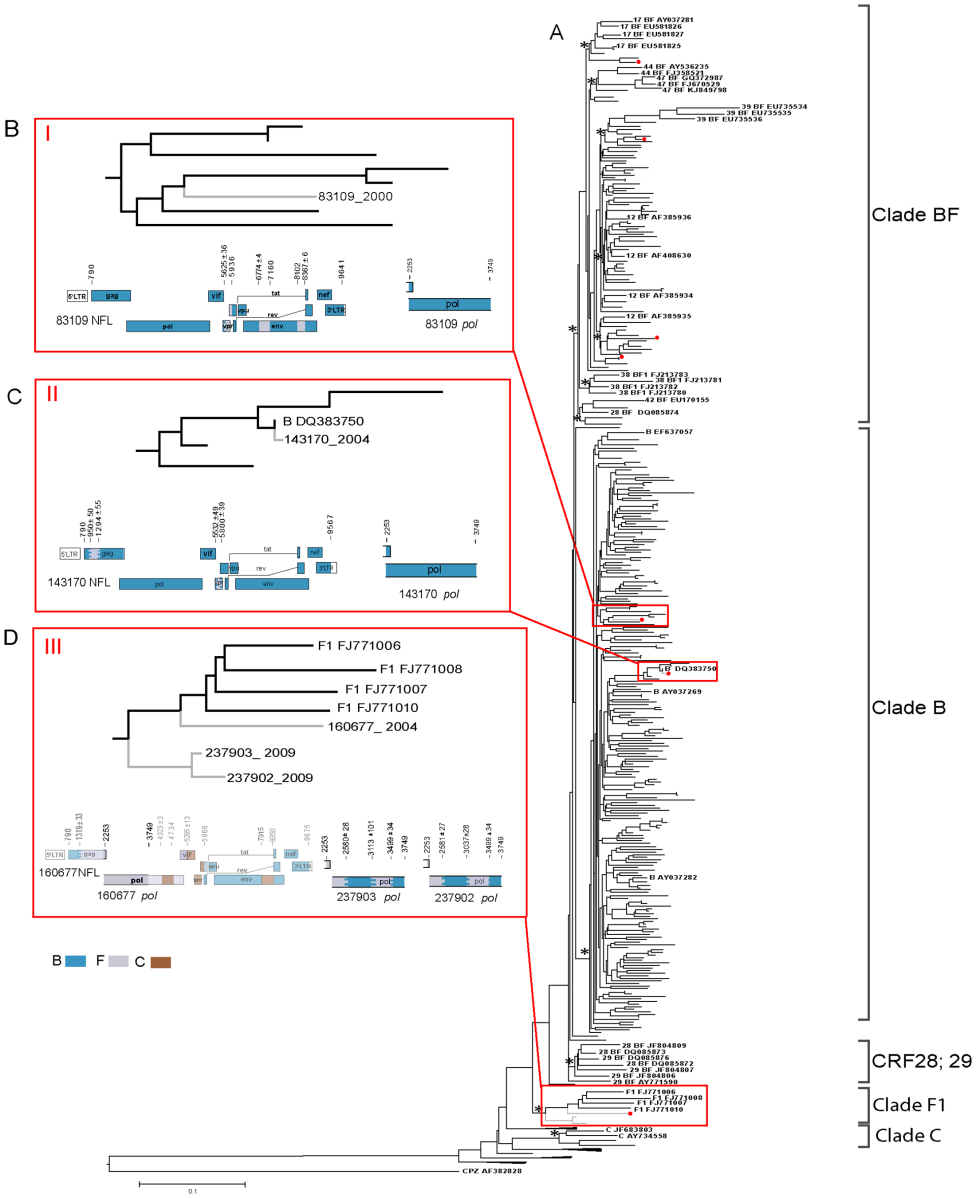
Phylogenetic tree of *pol* sequences and genetic mosaic for the recombinant sequences misclassified by phylogeny. (A) Maximum likelihood phylogenetic tree of the HIV-1 *pol* (1467 nt) sequences obtained from 312 Argentinean MSM. Reference sequences include BF recombinant and pure subtype sequences and the tree was rooted using SIVcpzGAB2 as outgroup. The branch support values are indicated with an asterisk (aLRT> 0.9) at key nodes. The seven *pol* sequences indicated with a red dot at terminal node were subsequently characterized as BF recombinant by means of NFL analysis. Red boxes are clusters of nucleotide sequences exhibiting discrepancies between *pol* gene- and NFL-based subtype assignment are zoomed in (Fig.1B), (Fig.1C), and (Fig.1D). Genetic mosaic maps for #83109 (box I) and #143170 (box II) pol sequences are consistent with phylogeny shown in Fig.1A but the NFL-sequence genetic mosaics exhibit as BF recombinants (Fig.1B and 1C, respectively). The #237902, #237903 *pol* sequences (box III) are phylogenetically ascribed to pure subtype F (Fig.1A) but their genetic mosaic exhibited them as BF recombinants. The #160677 *pol* sequence (box III) also phylogenetically classified as pure subtype F, shows a NFL-based genetic mosaic characterized as B-C-F recombinant (Fig.1D).

The phylogenetic relatedness after ML analysis showed 81 out of the 312 Argentine HIV-1 *pol* sequences clustering in CRF_BF clade, including 46 closely related to CRF12, 14 related with other CRFs_BF (CRF17, 28, 29, 39, 42, 44, 47), and 21 sequences -defined as URFs_BF- segregated in an independent clade. Other 2 sequences were closely related with subtype C, and 3 sequences grouped into subtype F clade. Additionally, 3 sequences did not group any of the major clades, and the remaining 223 sequences grouped into subtype B clade (Fig 1).

When investigating the level of intermixing of HIV-1 strains from MSM with those characterized previously from the Argentinean general population, *pol* gene sequences appeared intermingled. Furthermore, the phylogenetic relatedness of the HIV-1 *pol* sequences from MSM appeared intermingled along the BF clade independently of their recombination pattern. All these associations were sustained with high support values (Fig 1).

Interestingly, two out of three *pol* sequences ascribed previously to subtype F by phylogenetic analysis (#237902, #237903), appeared as a BF recombinant after recombination detection methods were applied. The remaining *pol* sequence (#160677) classified as subtype F was finally characterized as triple B/C/F recombinant when the NFL sequence was analyzed (Fig 1D).

**Fig 2:**
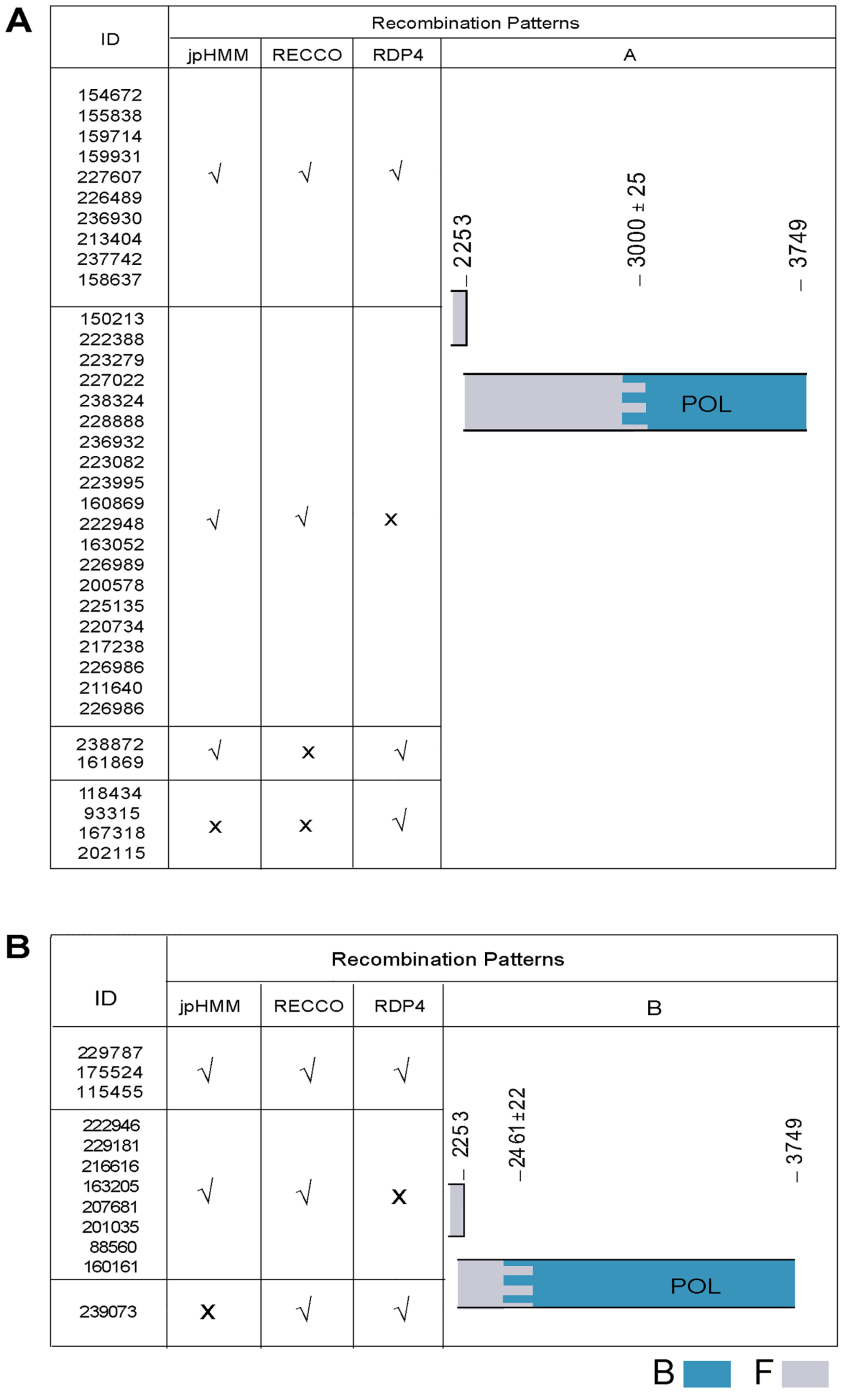
Predominant BF recombination patterns detected by different methods. Maps of BF recombinant genetic mosaic patterns (A and B) and breakpoint locations at *pol* gene. Nucleotide sequences named with their ID are grouped according to their concordance (√) or discrepancy (X) in breakpoint locations defined according to the three *in silico* methods used (jpHMM, RECCO, and RDP4).

The genetic mosaicism at *pol* gene was defined by using different *in silico* tools able to establish the breakpoint locations. Different recombination patterns were characterized. Two of them, named A and B, exhibited one breakpoint at different locations. These two breakpoints are present in the CRF12_BF pattern plus another one at position 2641. In patterns A and B, breakpoints were located at nucleotide position 3000±25 and 2461±22 respectively. The breakpoint in the pattern B is also present in CRF38_BF. Thirty-six sequences showed pattern A (Fig 2A), 12 exhibited pattern B (Fig 2B), 6 exhibited non-A non-B recombination patterns and, the remaining 32 sequences showed undefined mosaic patterns that were multiple but not associated with any of the previous patterns. Primary associated-resistance mutations (M184V and/or K103L) were detected in a minority of *pol* sequences ascribed to pattern A (n = 3) and B (n = 2).

**Fig 3:**
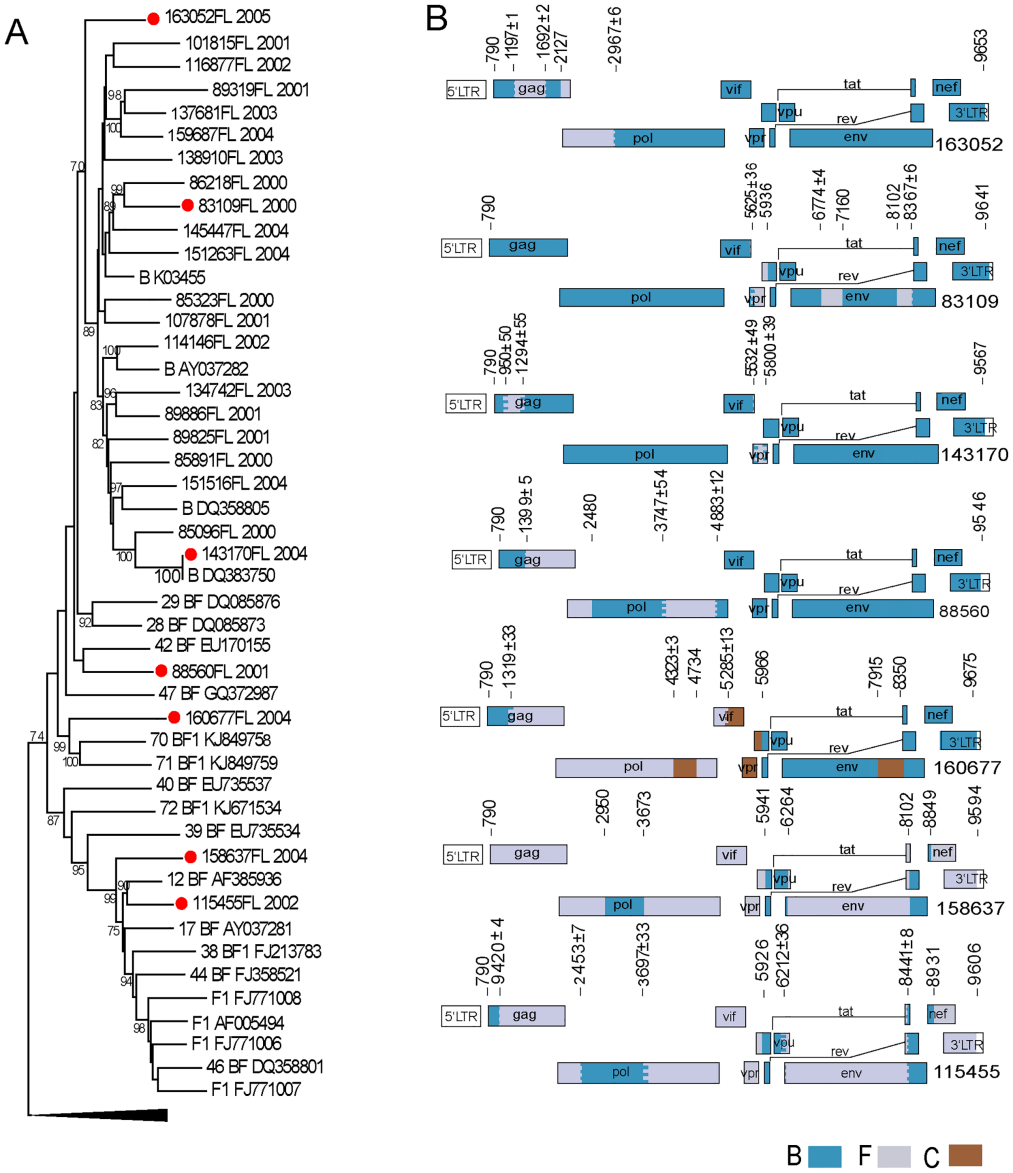
Phylogenetic tree of near full length sequences and recombination patterns corresponding to BF recombinant sequences. (A) Maximum likelihood phylogenetic tree of 25 HIV-1 NFL sequences obtained from Argentinean MSM. Those sequences characterized as BF recombinants once genetic mosaic was defined, are indicated with a red dot in the terminal node. Bootstrap values ≥ 70 are indicated in the nodes. (B) Maps of the genetic mosaic patterns including breakpoint locations of the 7 BF recombinant NFL sequences.

Phylogenetic relatedness between the 25 HIV-1 near full length (NFL) sequences depicted 4 sequences clustered within BFs recombinant clade. Of them, one was closely related to CRF12_BF and one to CRF42_BF reference sequences (#115455 and #88560 respectively), and the remaining two segregated alone (#158637 and #160677). The other 21 NFL sequences were clustered into the subtype B clade (Fig 3A).

When recombination breakpoints location was defined by *in silico* analysis, 7 out of 25 NFL sequences showed evidence of inter-subtype recombination. Coincidentally with phylogenetic inference, the sequence #115455 shared breakpoints compatible with CRF12_BF but, as previously mentioned, with pattern B at *pol* gene instead of pattern A. Similarly, the sequence #158637 depicts also similar breakpoints with CRF12 but exhibited the pattern A at *pol* gene. As mentioned above, sequence #160677 exhibited a genetic mosaic evidencing B-C-F triple recombinant. Sequence #88560 shared breakpoints with CRF29_BF (Fig 3B).

Interestingly, the other three sequences that characterized phylogenetically as subtype B appeared as BF recombinants. Sequences #83109 and #143170, classified previously as B by phylogeny and recombination detection analysis on *pol* gene fragments (Fig 1B and 1C, respectively) as well as #163052 sequence included subtype F fragments with dissimilar size (from 200-to-700bp) and genomic location (*gag*, *pol*, *vpr-env*) (Fig 3B). These recombination patterns did not match any of the CRF_BF described previously, and may represent plausible new URFs_BF.

### Concordance analysis

The concordance recombination detection and breakpoint positions between partial HIV-1 *pol* sequences and near-full length sequences data were analyzed in more detail by computing the level of agreement between the two sources of genomic evaluation. The relatively low kappa score indicates that the agreement was good (0.689±0.4 to 0.98; p<0.001) [34].

### Estimation of the time to the most recent common ancestor (tMRCA)

**Fig 4:**
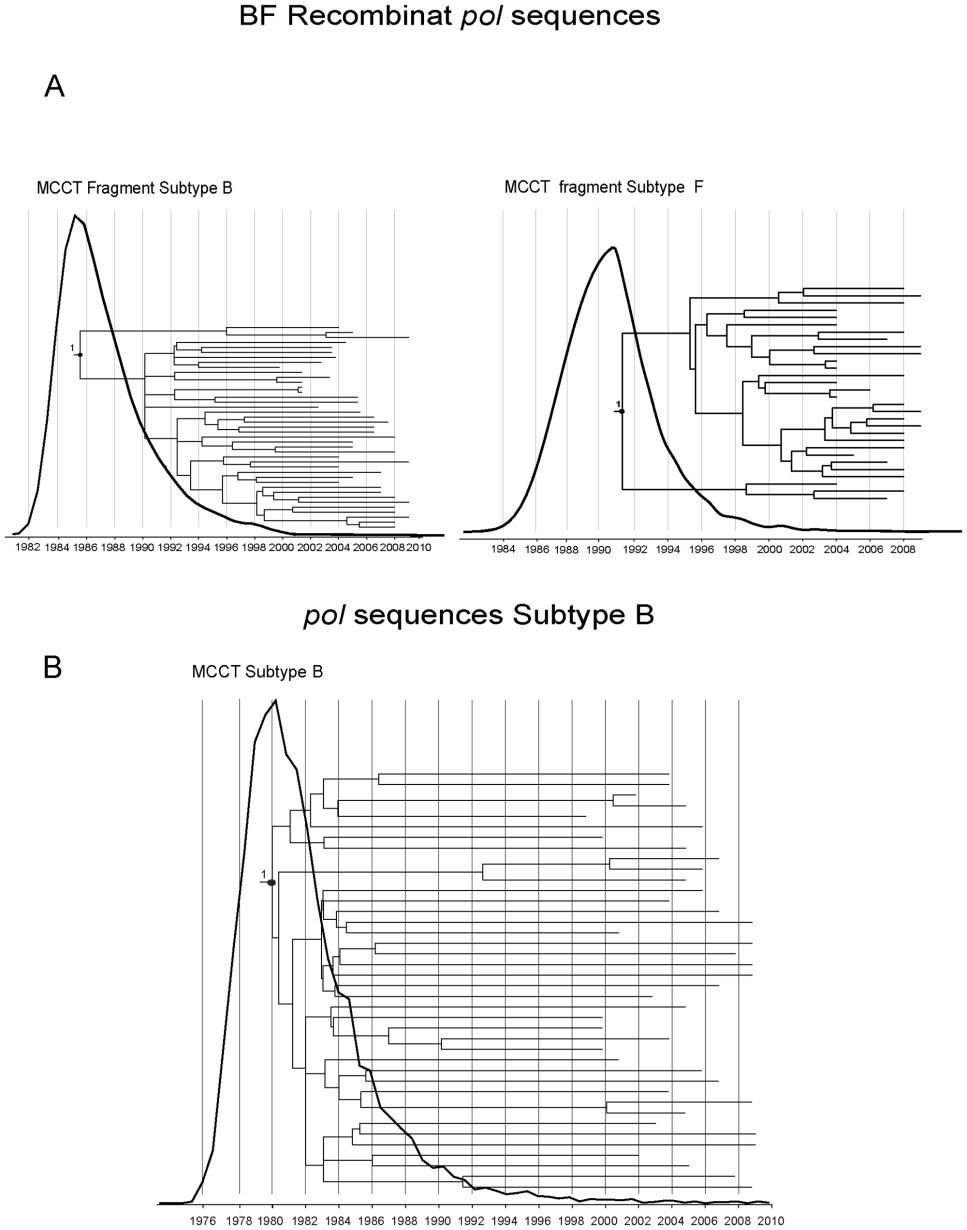
Bayesian analysis for BF recombinant sequences and pure B sequences from MSM population. Time-calibrated maximum clade credibility trees (MCCT) of HIV-1 BF and B clades identified in the *pol* ML analyses. (A) For BF recombinant sequences we obtained two MCCT corresponding to the analysis of pure subtype B and F fragments independently of each other. (B) MCCT obtained by analyzing the subtype B pure sequences. The posterior probabilities are indicated at key nodes (≥ 0.90). In each tree, the node indicating the most recent common ancestor of the MSM Argentinian B and F sequences fragments is indicated with a black circle.

Our analysis performed over pure subtype B fragment sequences from BF recombinants (nucleotide positions 3051 and 3719 according to HXB-2 numbering) found the maximum probability for the ancestor to that clade in the late 1980s (tMRCA: 1986; 95% HPD: 11.21–55.38) (Fig 4A). Almost coincidently, the estimation based on pure subtype F fragment sequences from BF recombinants (nucleotide positions 2253 and 2904 according to HXB-2 numbering) was performed in the early 1990s (tMRCA: 1991; 95% HPD: 8.66–36.31) (Fig 4A). Regarding pure subtype B sequences (nucleotide positions 2253 and 3293 according to HXB-2 numbering), the analysis found the maximum probability for the ancestor to that clade in the early 1980s (tMRCA: 1979; 95%HPD: 20.1851–46.9062) (Fig 4B). The substitution rates found were 3x10^-3^ (5.27x10^-4^–5.77x10^-3^), 2.9x10^-3^ (3.18x10^-4^–6.17x10^-3^) substitutions/site/year for the subtype B and F fragments from BF recombinant sequences and 1.839x10^-3^ (95%HPD: 9.6x10-4–2.6931x10^-3^) substitutions/site/year for the subtype B pure sequences, thus remaining within the order of magnitude of 10^−3^ expected for the HIV-1 *pol* gene [32, 33, 35, 36].

## Discussion

Here we performed for the first time an extensive *in-depth* analysis of the genetic diversity of HIV-1 strains circulating in the MSM population from Argentina. Such characterization implied HIV-1 *pol* gene and near full length sequences used for phylodynamic analysis and definition of the genetic mosaic structure.

In Argentina, HIV-1 BF recombinants and subtype B viruses were found with dissimilar prevalence according to the at-risk study population [8, 9, 19]. Firstly, BF recombinants have revealed the presence of three different recombination patterns after the analysis of 284 BF *pol* sequences from adult patients [16]. Recently, other recombinant structures have been described in two studies involving a small group of pregnant women, and a group of HIV-1 vertically-infected children [1, 18].

The present study addresses a larger population of Argentinean MSM allowing characterizing 337 HIV-1 sequences. Of them, CRF12_BF-like sequences were the most common form identified (>56%) by phylogenetic inference based on *pol* and near full-length sequences. In a lesser extent, other BF recombinant sequences characterized at *pol* gene appeared to be closely related with CRFs_BF reported previously in Argentina (CRF17_BF-like) [1, 14], as well as others unrecognized previously (CRF28, 29, 39, 42, 44, and 47) but reported in neighboring countries such as Uruguay [12], Brazil [10], and Chile [13]. This finding is plausible considering the fluent population exchange between these countries. Additionally, numerous recombinant sequences defined as URFs_BF were also found. Furthermore, BF recombinants were deeply characterized after defining the genetic mosaicism patterns using several recombination detection programs simultaneously. These *in silico* strategies showed an acceptable coincidence among each other and allowed a rigorous analysis of the HIV-1 recombinants but also denote plausible inconsistencies between them: location of breakpoints, minimum length of inserted genomic fragments able to be detected and phylogenetic uncertainty. The analysis of genetic mosaicism by multiple strategies suggests the presence of unrecognized BF recombination patterns allowing a deeper and unbiased molecular characterization. Nevertheless, the occurrence of recombination based on *pol* gene sequences exhibited a good degree of agreement when comparing with results based on NFL sequences, but a larger dataset is required to reach more conclusive results.

Taken together, these analyses depict a complex heterogeneity for HIV-1 BF recombinant forms, including the presence of gene mosaics sharing some -but not all- recombination breakpoints with well-defined CRFs_BF. Thus, new genetic mosaics showing larger subtype B segments appeared among MSM in Argentina. These variants may be considered as “second generation recombinants” originated by successive rounds of recombination occurred between the CRF12_BF and the subtype B circulating locally, as proposed previously [37].

Regarding the Bayesian analysis, our estimation of the tMRCA performed specifically over the BF recombinant sequences circulating in the MSM population showed a relatively recent coalescence time, consistent with previous assumptions [14]. The introduction of both B and F subtypes is probably a recent event dated in the end of 1980s-early 1990s. Hence, the CRFs_BF variants have been circulating among Argentinean MSM for about 30 years, although its presence was not detected until recently due to its lower prevalence compared with subtype B, which appeared to be introduced in the early 1980s. Previously it was reported that the tMRCA based on subtype B fragments from CRF12_BF recombinants was introduced in the early 1970s [38]. Such a difference may be explained by the diverse experimental designs used between this study and that by Dilernia *et al*. [38] included individuals from the whole population of infected individuals, whereas we focused only on MSM. In addition, the time of period encompassed by Dilernia *et al*. was broader than the period analyzed by us.

## Conclusion

The characterization of HIV-1 BF recombinant forms from MSM in Argentina revealed the presence of CRF12-like variants accompanied by a heterogeneous mix of strains exhibiting several mosaic patterns, including non-reported URFs. Their phylodynamic analysis showed transmission expansion and provides evidence of BF recombination as a dynamic and persistent phenomenon spreading among MSM in Argentina allowing the detection of active clusters as potential intervention targets to hinder HIV-1 transmission.
